# Spatial frequency equalization does not prevent spatial–numerical associations

**DOI:** 10.3758/s13423-022-02060-w

**Published:** 2022-02-07

**Authors:** Andrea Adriano, Luca Rinaldi, Luisa Girelli

**Affiliations:** 1 Dipartimento di Psicologia, Università degli Studi di Milano-Bicocca, Piazza dell’Ateneo Nuovo 1, Edificio U6, 20126 Milano, Italy; 2 Department of Brain and Behavioral Sciences, University of Pavia, Pavia, Italy; 3 Cognitive Psychology Unit, IRCCS Mondino Foundation, Pavia, Italy; 4 NeuroMI, Milan Center for Neuroscience, Milano, Italy

**Keywords:** Spatial frequency, Spatial–numerical association, Hemispheric asymmetries, Numerical processing

## Abstract

There is an intense debate surrounding the origin of spatial–numerical associations (SNAs), according to which small numbers are mapped onto the left side of the space and large numbers onto the right. Despite evidence suggesting that SNAs would emerge as an innate predisposition to map numerical information onto a left-to-right spatially oriented mental representation, alternative accounts have challenged these proposals, maintaining that such a mapping would be the result of a mere spatial frequency (SF) coding of any visual image. That is, any smaller or larger array of objects would naturally contain more low or high SF information and, accordingly, each hemisphere would be preferentially tuned only for one SF range (e.g., right hemisphere tuned for low SF and left hemisphere tuned for high SF). This would determine the typical SNA (e.g., faster RTs for small numerical arrays with the left hand and for large numerical arrays with the right hand). To directly probe the role of SF coding in SNAs, we tested participants in a typical dot-arrays comparison task with two numerical sets: one in which SFs were confounded with numerosity (Experiment 1) and one in which the full SF power spectrum was equalized across all stimuli, keeping this cue uninformative about numerosity (Experiment 2). We found that SNAs emerged in both experiments, independently of whether SF was confounded or not with numerosity. Taken together, these findings suggest that SNAs cannot simply originate from SF power spectrum *alone*, and, thus, they rule out the brain’s asymmetric SF tuning as a primary cause of such an effect.

**Supplementary Information:**

The online version contains supplementary material available at 10.3758/s13423-022-02060-w.

In a seminal paper published almost 30 years ago, Dehaene et al. (1993) reported for the first time that adult participants tested in a parity judgment task of symbolic digits were faster to respond to small numbers with the left hand and to large numbers with the right hand. This effect has been taken as an empirical proof supporting the intuitive idea that numbers are spatially organized from left to right along a mental line (Galton, 1880a, b) and, beyond being replicated in various settings (e.g., Cipora et al., 2019), this phenomenon has inspired much subsequent work (de Hevia et al., 2008). Although originally reported with symbolic stimuli (e.g., Arabic digits or number words), similar spatial–numerical associations (SNAs) have been obtained also with nonsymbolic numerical stimuli (e.g., arrays of objects or sequences of tones) allowing replication in animal research (e.g., Rugani et al., 2015, 2020) and in preliterate children (Bulf et al., 2016; de Hevia et al., 2014; de Hevia et al., 2017; Ebersbach et al., 2014), hence suggesting a biological foundation of this mapping. Accordingly, recent works employing nonsymbolic dot-arrays comparison tasks in adult participants reported the presence of SNAs compatible with those observed in infants and animals (Nemeh et al., 2018; Zhou et al., 2016). For instance, Zhou et al. (2016) used a same/different matching task to investigate the spatial representation of nonsymbolic numerosity, controlling also for low-level visual features such as size and density typically confounded with numerosity. Results showed that neither size nor density affected responses, yet results yielded faster right-hand responses to large nonsymbolic numerosities only (no difference was found for left-hand responses). Furthermore, Nemeh et al. (2018), using a target-to-reference comparison task, showed typical SNAs congruency effect (although in this case arrays were not controlled for any low-level feature).

Hence, the presence of SNAs in nonhuman animals and in humans, even at birth, has been mainly traced back to biological determinants. These include the shared representation of numerical and spatial information at the neural level (Hubbard et al., 2005), as well as the early biases in the control of visuospatial attention, such as the tendency to overattend, and start scanning from, the left side of space induced by the right hemisphere dominance for spatial processing (de Hevia et al., 2012). The early predisposition to map numbers onto space would be later modeled by experiential factors, such as reading habits, which would modulate the direction of SNAs (de Hevia, 2021).

Yet a very recent theoretical proposal—the *brain’s asymmetric frequency tuning* (BAFT) hypothesis—suggested that SNAs would simply reflect laterality differences in the way the brain processes specific physical features in the actual numerical stimuli (Felisatti et al., 2020a, b). In particular, this hypothesis assumes that SNAs would emerge as the result of brain asymmetries relative to the processing of the raw spatial frequencies (SF) content naturally correlated with dot numerosity stimuli (or any other visual image), with the left or right brain hemisphere preferentially dedicated to process high or low SF bands, respectively. SFs are generally defined as the number of dark/light cycles per degree of visual angle (or per image). Low SFs (few cycles per degree) capture the global distribution of light and dark across the entire scene; high SFs (many cycles per degree) instead code local changes from light to dark that correspond to smaller elements (e.g., De Valois & De Valois, 1990). Since small numerical arrays of dots would contain fewer local changes from light to dark (e.g., edges), they would be ideally represented by SF-defined contrast gratings with a few large strips per degree (e.g., low SF spectrum), while large arrays would contain more local dark/light variations and would be ideally represented by grating with many thin strips per degree (e.g., high SF spectrum). Hence, nonsymbolic SNAs would be the result of the lateralized SF processing in each hemisphere. For example, in the case of adult participants, a visually presented small array of dots would engage more the right hemisphere, inducing in turn a left bias and speeding up the manual response with the left hand (Felisatti et al., 2020a, b). Accordingly, since in the study of Nemeh et al. (2018) dot size was constant, SFs information were strictly correlated with dot numerosity, leaving open the possibility that their results could be explained by the BAFT account.

Crucially, the BAFT account makes very specific testable experimental predictions since it assumes that, overall, SNAs are driven by the physical content of the stimuli such as their SF power spectrum (e.g., De Valois & De Valois, 1990). This means that when this physical information is not informative about numerosity depicted in the stimuli, SNAs should not be observed (e.g., Wichmann et al., 2010). Here, to directly test this hypothesis, we run two experiments on adult participants: In Experiment 1, they were required to perform a typical sequential nonsymbolic comparison task (Nemeh et al., 2018) with classic arrays of dots (i.e., original stimuli), but controlled for five main low-level features. In striking contrast, in Experiment 2 we removed SF information as a cue for numerosity by equalizing the full spectrum across all numerical dot arrays (see also Adriano et al., 2021a, b). According to the BAFT hypothesis, if SNAs originate from the brain asymmetrical tuning in processing raw SF content, SNAs effect should be found with the original stimuli, but not with the SF-equalized ones. Alternatively, if SF information does not play a key role, we should expect spatial–numerical compatibility effects in both experiments, as well as a typical ratio effect (e.g., Whalen et al., 1999).

## Experiment 1: Comparison task with original stimuli

In Experiment 1, we tested whether nonsymbolic dots arrays triggered SNA using a classic dots comparison task. Participants were presented with a stimulus (reference) followed by a second one (test), and they had to decide whether the latter was numerically smaller or larger than the reference (for a similar task, see Nemeh et al., 2018). Unlike previous studies that did not fully control low-level features correlated with numerosity (Nemeh et al., 2018; Zhou et al., 2016), here, we used dot arrays controlled for five main visual features: convex hull, total surface, density, item size, and total circumference (Gebuis & Reynvoet, 2011). We manipulated the numerical ratio between reference and test stimuli and the mapping of response keys: In one condition, the mapping was congruent (e.g., “smaller” was associated with a left response and “larger” with a right response), whereas in the other condition it was incongruent. We predicted that if SNAs emerge from spatial coding of nonsymbolic numerical information, we should observe a typical ratio effect and a typical congruency mapping effect (e.g., slower RTs for incongruent mapping).

### Materials and methods

#### Participants

We performed an a priori power analysis with G*Power 3.1 (Faul et al., 2009) to determine our needed sample size. Because our study was inspired by the work of Nemeh et al. (2018), who found a large effect size (e.g., η_p_^2^ = .19) for the typical SNA congruency effect (e.g., Hand × Magnitude interaction), we assumed a standard large effect size (η_p_^2^ = .14) for our main variable of interest (e.g., congruency mapping). The calculation established that to obtain a large effect size (η_p_^2^ = .14) with an 80% of power for the main effect of the mapping (congruent vs. incongruent), in a one-way repeated-measures analysis of variance (ANOVA; two levels of measurements; alpha = .05), a minimum sample of 51 participants was required.^1^

A sample of 52^2^ undergraduate students from the University of Milano-Bicocca were recruited (41 females, 44 right-handed). The mean age was 22.53 years (*SD* = 3.69). Due to COVID-19 restrictions, participants performed the study online through the Pavlovia/PsychoPy platform (www.pavlovia.org). All participants had normal or corrected-to-normal vision and were unaware of the purpose of the experiment. Each participant signed an online informed consent document before the experiment began, and the study was conducted in accordance with the Declaration of Helsinki. The study was approved by the Local Ethical Committee (protocol N° RM-2020-230).

#### Stimuli

**Fig. 1:**
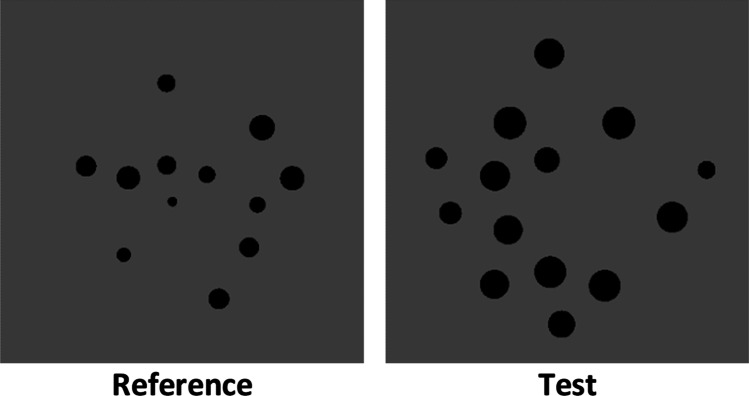
Example of original stimuli used in Experiment 1 as generated with the method of Gebuis and Reynvoet (2011)

Original stimuli were generated off-line with the script from Gebuis and Reynvoet (2011), which provides statistical controls over the following low-level visual cues: area extended (or convex hull), total surface (the aggregate surface of all dots in one array), density (area extended/total surface), item size (average diameter of the dots presented in one array), and total circumference (circumference of all dots in one array, taken together). Post hoc analyses ensured the absence of a relationship between numerical distance and the difference in visual properties (all *R*^2^ values < .05; see also the Stimuli Visual Parameters Analysis section in the Supplementary Materials). Each stimulus was composed of black dots of a random size (RGB = 0, 0, 0) depicted on a middle grey background (RGB = 127, 127, 127) and scattered across a squared stimulus panel (395 × 395 px; see Fig. 1). A total of 192 stimuli were generated (96 stimuli pairs). In each pair of stimuli, one set always contained 12 dots (reference), whereas the second numerosity (test) was smaller than 12 in half of the trials (8, 9, or 10 dots) and larger than 12 in the other half (14, 16, or 18 dots), resulting in three symmetrical ratios (smaller numerosity/larger numerosity: ratio 0.66, ratio 0.75, and ratio 0.8) around the reference numerosity. A total of 6 different relative comparisons between test and reference were generated in the stimuli: 8 vs. 12 (ratio 0.66), 9 vs. 12 (ratio 0.75), 10 vs. 12 (ratio 0.8), 12 vs. 14 (ratio 0.8), 12 vs. 16 (ratio 0.75), and 12 vs. 18 (ratio 0.66). For each of the six numerical ratios, the script generated 16 pairs with different spatial patterns.

#### Procedure

**Fig. 2:**
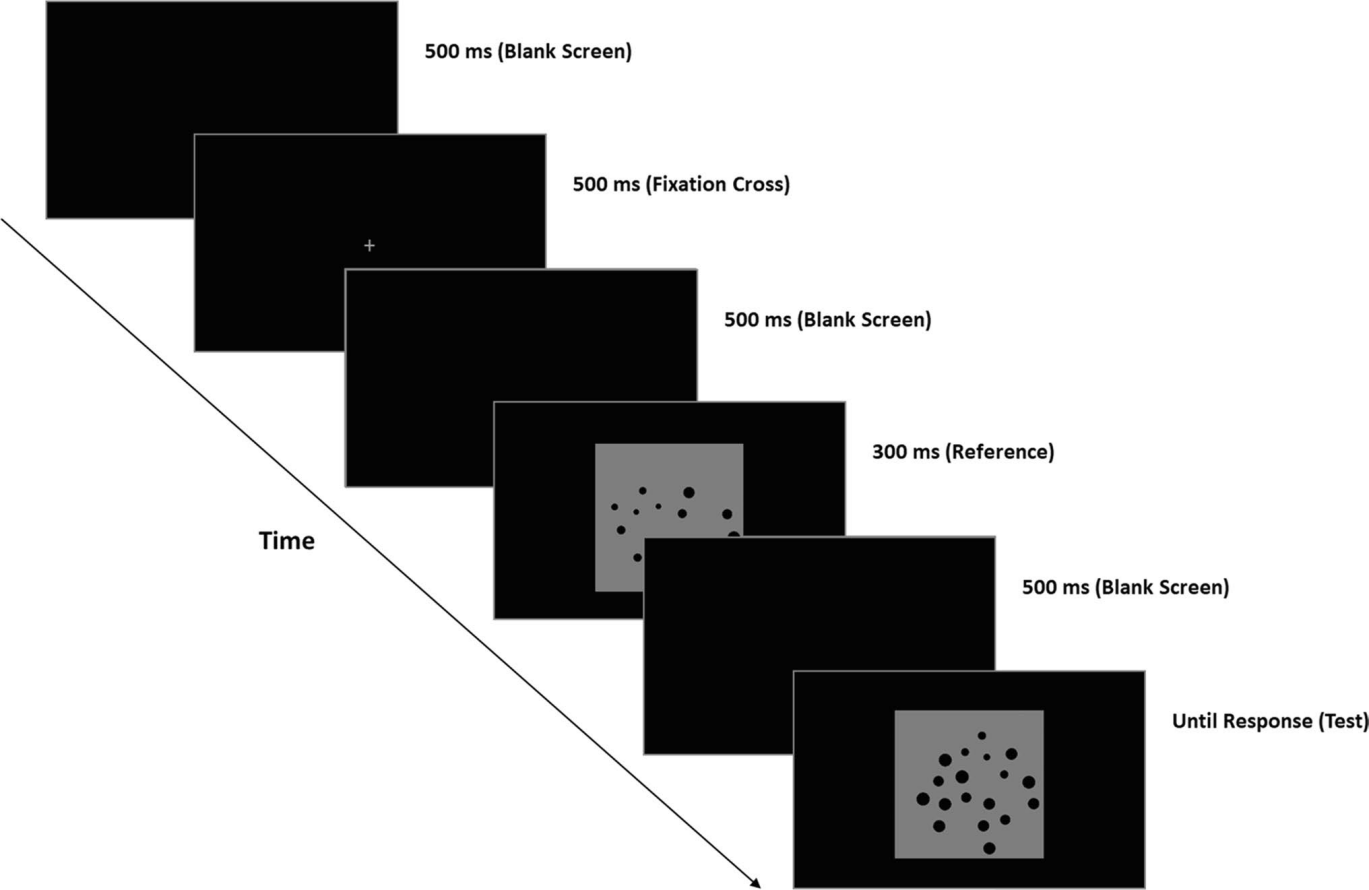
The number comparison task. The participant had to decide whether the test stimulus was numerically larger or smaller than reference stimulus. A total of 192 trials (96 trials × 2 blocks) were displayed.

Instructions and experimental stimuli were projected by means of an online PsychoPy routine (Peirce, 2007). The experimental task was a number comparison between two sequentially presented arrays of dots (e.g., to determine whether the test stimulus is numerically larger or smaller than the fixed reference). The experiment was preceded by a brief training period composed of eight trials allowing participants to familiarize with the task. In the training phase, we presented only the condition with the lowest (i.e., easiest) ratio (e.g., 0.66). Each trial started with a blank screen for 500 ms (RGB = 0, 0, 0), before a grey fixation cross (Font: Times; Size: 16 pixels; RGB = 127, 127, 127) was presented for an additional 500 ms and followed by a further blank screen (500 ms). Next, test stimulus was displayed within a black window (RGB = 0, 0, 0) on the screen centre for 300 ms; afterwards, a blank screen (500 ms) was presented until the onset of test stimuli, which stay on the screen until response (see Fig. 2).

After the training phase, two experimental blocks composed of 96 randomized trials were presented, for a total of 192 experimental trials. In one block (congruent mapping), participants were instructed to press the left key (“A” key) with their left index finger if they judged the test numerosity to be smaller than reference or to press the right key (“L” key) with their right index finger if they judged the test numerosity to be larger than reference. In the other block, the mapping of the keys was reversed (incongruent mapping). The order of blocks was counterbalanced across participants. Within a block, each of the six comparison pairs (test vs. reference) were repeated 16 times, resulting in 96 total trials per block (16 trials × 6 comparison pairs).

### Results and discussion of Experiment 1

**Fig. 3:**
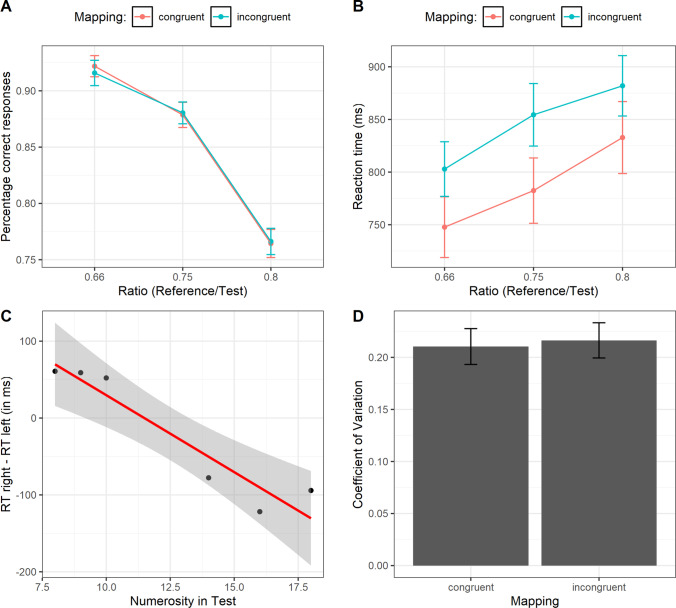
**a** Percentage of correct responses as a function of the absolute ratio and the mapping condition. **b** Reaction times as a function the absolute ratio and the mapping condition. **c** RT difference between responses with the right and left hands as a function of the numerosity in test stimuli. Shaded regions represent the 95% CI of the regression line. **d** Coefficient of variation for each mapping condition. Bars represent ±1 *SEM*

Two separated 2 × 3 repeated-measures ANOVAs were performed, with response mapping (congruent vs. incongruent) and numerical ratio (0.66, 0.75, 0.8) as within-subjects factors and with RTs or accuracy (percentage of correct responses) as dependent variable, respectively.^3^ The analyses of accuracy data only showed a significant main effect of numerical ratio, *F*(2, 102) = 187.11, ε = .89, *p* < .001, η_p_^2^ = .78, suggesting that discrimination was harder for higher ratios (see Fig. 3a). A post hoc test (Bonferroni correction) revealed a significant difference between the ratio 0.66 and the ratio 0.75, *t*(102) = 4.774, *p* < .001, *d =* .66, the ratio 0.66 and the ratio 0.80, *t*(102) = 18.622, *p* < .001, *d =* 2.58, and the ratio 0.75 and the ratio 0.80, *t*(102) = 13.84, *p* < .001, *d =* 1.9. On the contrary, no significant main effect of mapping, *F*(1, 51) = .009, *p* = .92, η_p_^2^ = .001, or interaction was found, *F*(2, 102) = .57, *p* = .52, η_p_^2^ = .003.

The RTs analysis on correct responses (data were log-transformed and trials outside ±1.5 times the interquartile range of distribution were eliminated, for a total of 3.14% datapoints removed) showed a significant main effect of numerical ratio, *F*(2, 102) = 41.2, ε = .88, *p* < .001, η_p_^2^ = .44, with numerosity discrimination becoming slower for harder ratios (see Fig. 3B). Post hoc comparisons (Bonferroni correction) revealed a significant difference between the ratio 0.66 and 0.75, *t*(102) = −4.772, *p* < .001, *d = *.66, the ratio 0.66 and the ratio 0.80, *t*(102) = −9.075, *p* < .001, *d =* 1.25, and the ratio 0.75 and the ratio 0.80, *t*(102) = −4.303, *p* < .001, *d =* .59. Crucially, we also found a significant main effect of mapping, *F*(1, 51) = 11.03, *p* = .002, η_p_^2^ = .178, with faster responses for congruent compared with incongruent mapping. No significant interaction was found, *F*(2, 102) = .98, ε = .88, *p* = .36, η_p_^2^ = .019.

To corroborate these results, we also run a further analysis following Fias et al. (1996). Reaction times with left-hand responses were subtracted from those with the right hand and were fitted with a linear regression as a function of each numerosity tested (Fias et al., 1996). Results showed that numerosity explained a significant proportion of variance in the differential RTs (e.g., right–left), *R*^2^ = .90, *F*(1, 4) = 35.1, *p* < .001. As expected, we found a significant negative regression coefficient, *β = −*.02, *t*(4) = −5.92, *p* < .001, suggesting a linear mapping: For numerosities smaller than reference, RTs were faster for the left hand and vice versa for numerosities larger than reference (see Fig. 3c; see also Supplementary Materials for the individual analyses, which are in line with the results reported here).

As a further independent index of numerical acuity, we also calculated the Coefficient of Variation (CoV) for each participant and mapping condition, as an index of the Weber fraction (e.g., Halberda & Odic, 2014). Gaussian cumulative distribution functions were fitted to the data (e.g., proportion of test stimuli correctly judged as more numerous than the reference, as a function of numerosity in test stimuli) and parameters were estimated with a parametric approach based on the maximum likelihood method, using Quickpsy package for R (Linares & López-Moliner, 2016). Psychometric curves were fitted considering the typical lapse in performance (e.g., missing a trial, finger errors) by allowing the value of the guess rate (γ) and lapse rate (λ) parameters to vary in the default range of 0–0.05 (Wichmann & Hill, 2001). The CoV was computed as the ratio between the standard deviation (*SD*) and the mean (e.g., point of subjective equality; PSE) of the psychometric functions (e.g., Helbig & Ernst, 2007). In line with the overall results of accuracy, we did not find a significant difference in the CoV between congruent and incongruent conditions, *t*(51) = −.324, *p* = .74, *d =* .045 (see Fig. 3d). Frequentist analyses were accompanied by Bayesian statistics that confirmed these results (see Supplementary Materials).

## Experiment 2: Comparison task with spatial frequency equalized stimuli

In Experiment 2, we specifically tested whether the SNAs merely emerge from the raw SFs content of the stimuli as predicted by the BAFT hypothesis. Participants were tested in a comparison task as in Experiment 1, but in this case, stimuli were equalized for SF content. According to the BAFT hypothesis, when this information is removed as cue for numerosity (e.g., it was equalized across all stimuli), we should expect no ratio effect and, crucially, no SNA effect (Felisatti et al., 2020a, b). That is, since all reference and test stimuli have the same power spectrum, participants cannot use this cue during the task to classify test stimuli as “larger” or “smaller” than the reference: hence, the performance should be merely at chance, showing no difference across mapping conditions (e.g., RTs for left-hand responses should be equal for smaller *and* larger numerosities, with such pattern extending to RTs for the right-hand responses). On the other hand, if SNA does not depend on SF content alone, the compatibility effect should still emerge (e.g., RTs for left-hand responses should be faster for smaller numerosities and slower for larger numerosities, while the opposite pattern should be observed for the right-hand responses).

### Materials and methods

#### Participants

A new sample of 52^4^ undergraduate students from the University of Milano-Bicocca were recruited (42 females, 50 right-handed). The mean age was 22.34 years (*SD* = 2.74). All the participants performed the study online through the Pavlovia/PsychoPy platform.

#### Stimuli and procedure

**Fig. 4:**
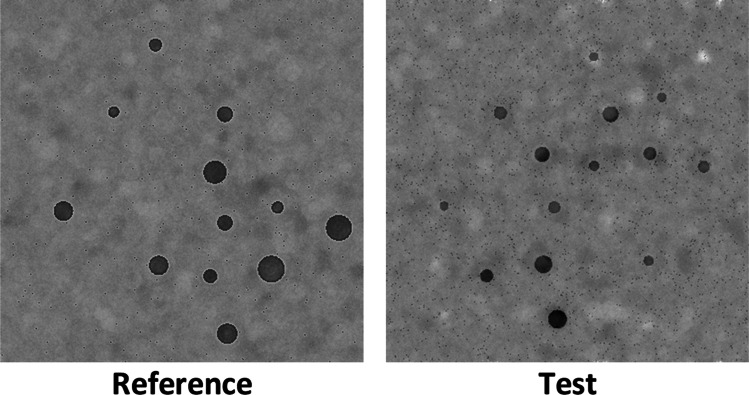
Example of SF equalized stimuli used in Experiment 2 as generated with the method of Willenbockel et al. (2010)

**Fig. 5:**
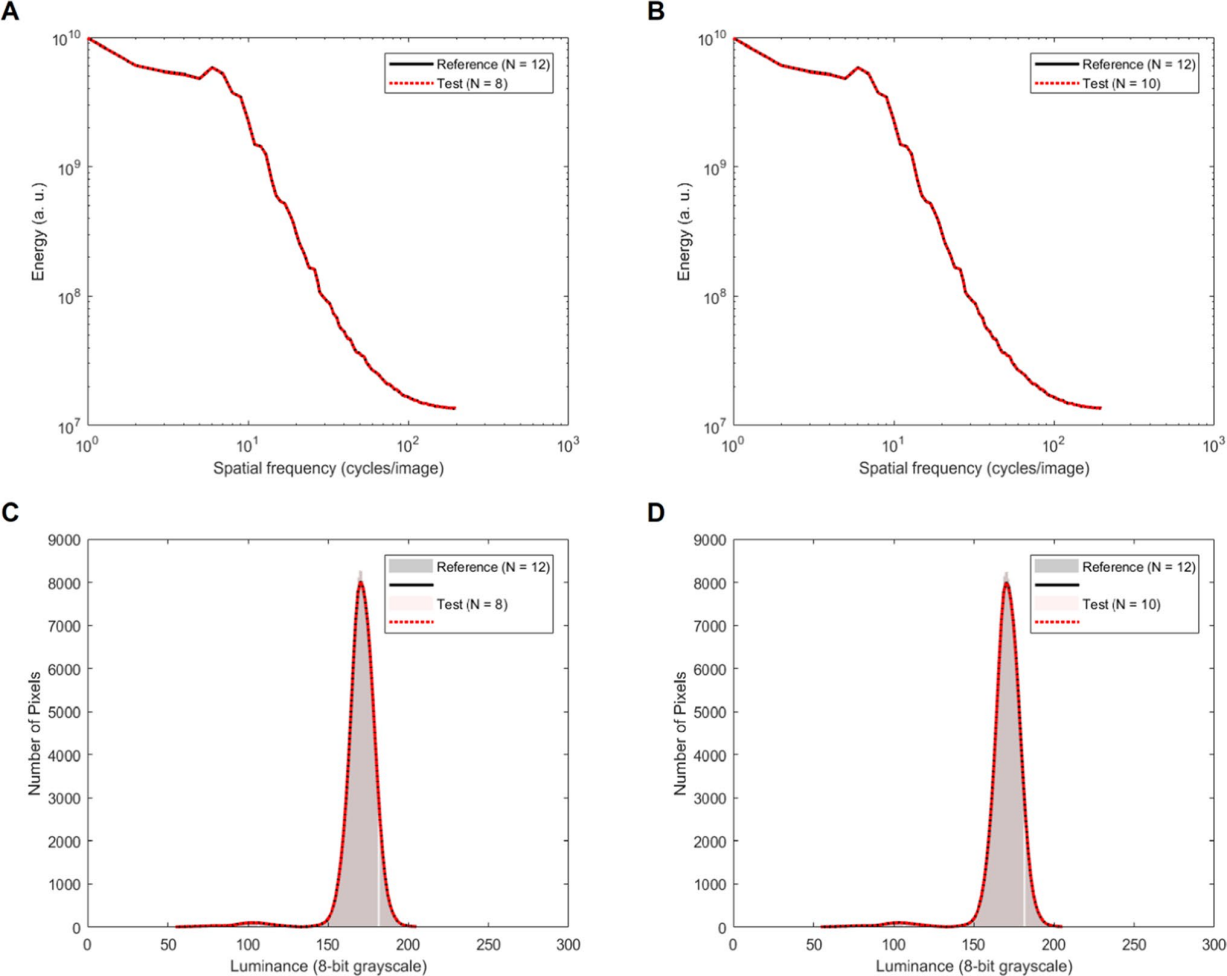
Rotational average of the Fourier energy spectrum (**a** and **b**) and luminance histogram profile (**c** and **d**) for two stimuli comparisons with different ratios (0.66 and 0.8), as presented in Experiment 2. Panels **a** and **c** show the low-level feature statistics for the test stimuli with eight items compared with the Reference (ratio 0.66), whereas Panels **b** and **d** show the low-level feature statistics for the test stimuli with 10 items compared with the Reference (ratio 0.8). Note that in all figures the curve profiles almost fully overlap, thus indicating an extremely high equalization of the low-level statistical properties of the stimuli. Stimuli images were coded in linear RGB 8-bit grayscale values

The procedure was identical to Experiment 1. The only difference is that original stimuli where postprocessed using a MATLAB script, following a similar methodology of Adriano et al. (2021a, b). Specifically, visual low-level statistical properties such as power spectrum and luminance histograms were fully equalized by processing all the original experimental stimuli with the SHINE toolbox for MATLAB (Willenbockel et al., 2010), which allows to match both the whole Fourier amplitude spectrum and the luminance histogram across each of the input images (see Fig. 4), preserving the original phase of each stimulus. In short, the average spectrum obtained across all input images was recombined with the original phase of each input stimulus (Wichmann et al., 2010). The complete set of 192 original stimuli (96 pairs) was submitted to an iterative algorithm (30 reiterations) to jointly match luminance histograms (*histMatch function*, which matches mean luminance, contrast, skew, etc.) and Fourier amplitude spectra (*specMatch function*, which matches SF and orientations). As can be observed in Fig. 5 and Fig. S1, indeed, each stimulus had a similar SFs amplitude spectrum and luminance profiles across numerosities and ratios.

### Results and discussion of Experiment 2

**Fig. 6:**
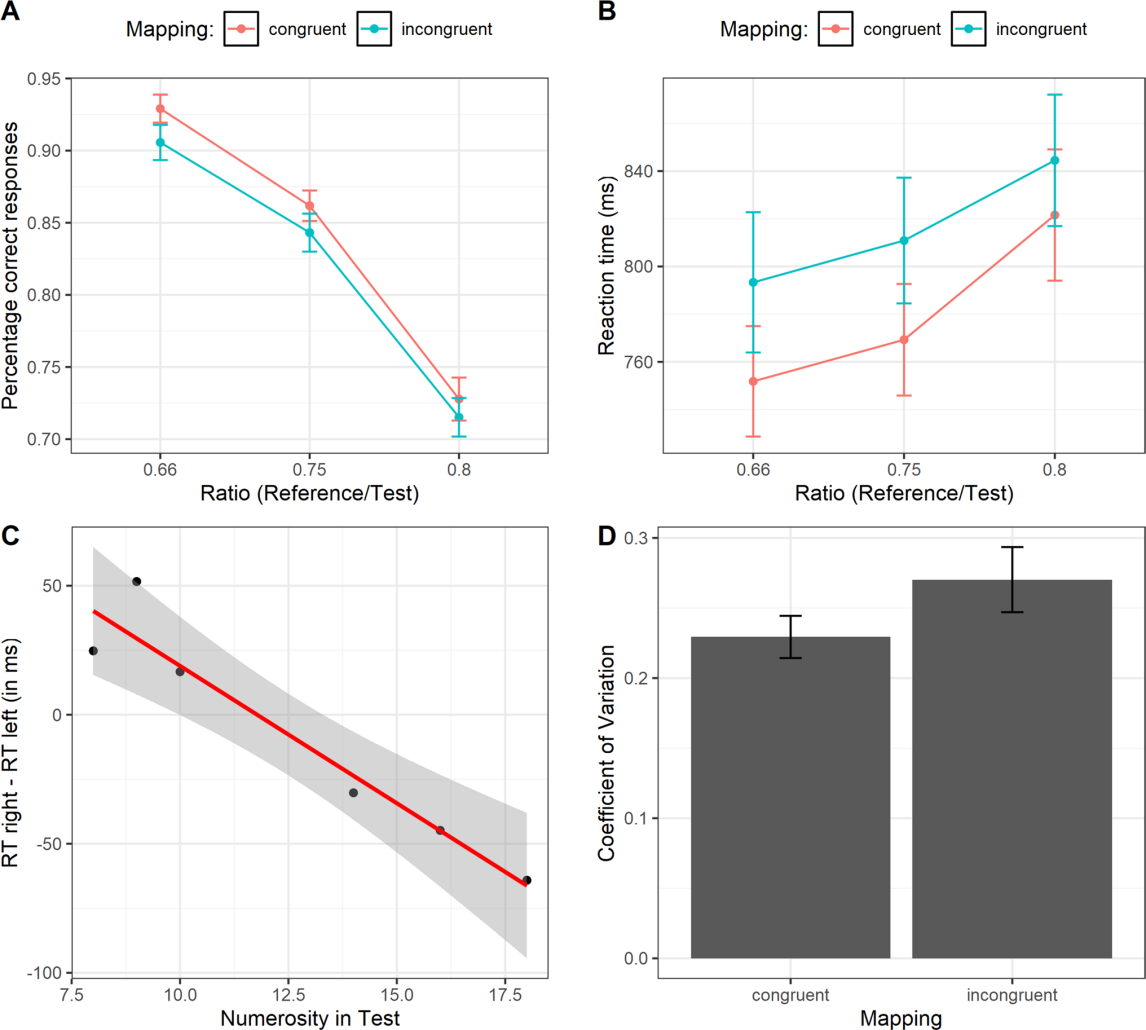
**a** Percentage of correct responses as a function of the absolute ratio and the mapping condition. **b** Reaction times as a function the absolute ratio and the mapping condition. **c** RT difference between right-hand and left-hand responses as a function of the numerosity in test stimuli. Shaded regions represent the 95% CI of the regression line. **d** Coefficient of Variation for each mapping condition. Bars represent ±1 *SEM*

Data were analyzed as in Experiment 1. We found a significant main effect of the ratio, *F*(2, 102) = 207.92, ε = .88, *p* < .001, η_p_^2^ = .80, Fig. 6A, and a significant main effect of mapping over accuracy, *F*(1, 51) = 5.9, *p* = .019, η_p_^2^ = .104, but no significant interaction, *F*(2, 102) = .238, ε = .83, *p* = .74, η_p_^2^ = .005. Post hoc (Bonferroni correction) revealed a significant difference between the ratio 0.66 and the ratio 0.75, *t*(102) = 6.631, *p* < .001, *d =* .92, the ratio 0.66 and the ratio 0.80, *t*(102) = 20.01, *p* < .001, *d =* 2.7, and between the ratio 0.75 and the ratio 0.80, *t*(102) = 13.38, *p* < .001, *d =* 1.85.

Analysis of RTs (4.65% of data were discarded) showed a main effect of ratio, *F*(2, 102) = 43.22, *p* < .001, η_p_^2^ = .45. Post hoc comparisons (Bonferroni correction) revealed a significant difference between the ratio 0.66 and the ratio 0.75, *t*(102) = −3.01, *p* = .010, *d =* .41, the ratio 0.66 and the ratio 0.80, *t*(102) = −9.12, *p* < .001, *d =* 1.26, and between the ratio 0.75 and the ratio 0.80, *t*(102) = −6.11, *p* < .001, *d =* .84. Crucially, a significant main effect of mapping was also found, *F*(1, 51) = 4.57, *p* = .037, η_p_^2^ = .082, see Fig. 6b, with faster RTs for the congruent mapping as compared with the incongruent one.^5^ No significant interaction was found, *F*(2, 102) = .72, *p* = .48, η_p_^2^ = .014.

Finally, we replicated the regression analysis with the RTs difference between right-hand and left-hand responses as dependent variable, and numerosity as predictor. Again, we found that numerosity explained a significant proportion of variance in RTs, *R*^2^ = .93, *F*(1, 4) = 52.99, *p* = .001, with a significant decreasing regression coefficient, *β= −*.01, *t*(4) = −7.28, *p* = .001, suggesting a linear mapping (see Fig. 6c; see also Supplementary Materials for the individual analyses). As a further metric of numerical precision, we calculated the CoV (an index of the Weber fraction) for each mapping condition. We found that in the congruent condition participants presented also a slightly better precision (smaller CoV) compared with the incongruent condition, *t*(51) = −2.25, *p* = .029, *d* = .31, which is in line also with higher accuracy found for the congruent condition compared with the incongruent condition. Frequentist analyses were accompanied by Bayesian statistics that confirmed these results (see Supplementary Materials).

## General discussion

In this study, we directly tested the *brain’s asymmetric frequency tuning* hypothesis (Felisatti et al., 2020a, b), probing whether nonsymbolic spatial–numerical associations originate from a mere spatial frequency coding of the raw visual input. Results from two experiments revealed the presence of a spatial–numerical association with nonsymbolic numerosity information regardless of whether spatial frequencies were equalized or not. Indeed, in Experiment 2, we completely ruled out the role of SF, since both the spatial congruency mapping and ratio effect were replicated when the full power spectrum was equalized across all stimuli. According to this hypothesis, any smaller/larger numerosity would be naturally associated with lower/higher SF content, and this would determine the observed spatial mapping (as the left/right hemisphere preferentially processes higher/lower SF information). In other terms, the BAFT theory provides a strong neurological explanation of the spatial–numerical association effect, but it represents a general visual mechanism for the processing of any visual image, including numerosity. Accordingly, the presence of SNAs across development in preliterate children (Bulf et al., 2016; de Hevia et al., 2014), in human adults (Nemeh et al., 2018; Zhou et al., 2016), as well as in animals (Rugani et al., 2015, 2020), particularly in studies using dot stimuli, is assumed to be the mere result of the lateralization of neural structures devoted to process SFs information composing any visual image. For example, in human adults the right hemisphere should be tuned for low spatial frequencies and, accordingly, stimuli with low SF information would be preferentially processed by this part of the brain, that also control for the contralateral left hand, inducing a left-bias (and vice versa for the left hemisphere). Thus, according to BAFT account, since the right hemisphere is tuned for low SF, the speed of response with the (contralateral) left hand should be fastened depending on the low SF content of a visual image presented at the center of the visual field. This should explain why visually presented small numerical arrays of dots (e.g., low SF information) should be judged faster with the left hand, compared with larger arrays of dots (e.g., high SF information). However, in Experiment 2 all the numerical test stimuli were matched for the whole power spectrum, which means that physical information reaching the right hemisphere was constant across all numerical arrays and mapping conditions. Hence, with power spectrum equalized stimuli under the BAFT account hypothesis, the reaction times for left-hand responses should have been similar for stimuli depicting larger or smaller numerosities, since both visual sets contained the same (low) raw SFs information amount. Yet, and contrarily to BAFT predictions, in Experiment 2 we specifically found that latencies for left-hand responses were still modulated by the *numerical content* depicted in the arrays (encoded in the original phase of the stimuli), rather than by the raw power spectrum. Indeed, we found a typical congruency mapping, which means that RTs for left hand were faster for smaller numerosities and slower for larger numerosities (and vice versa for the right hand). While several models have been proposed to explain SNAs (for a review, see Van Dijck et al., 2015), the BAFT model is perhaps the most reductionist among them, since it does not assume any particular cognitive (e.g., numerical mental line representation) or attentive factor behind the whole process and is rather rooted on a strict number of computational factors: the lateralization of brain structures processing different SFs ranges and, crucially, the physical information contained in the raw visual input stimulus. In a neuro-computational metaphor, any algorithm trained to classify numerical stimuli (e.g., as larger or smaller) extracting *only* their raw power spectrum, would be “tricked” if tested with our SF-spectrum equalized stimuli (Wichmann et al., 2010) and would simply fail to do the task. If our brain implements a similar processing mode, real observers should have failed completely the comparison task, and no SNA and ratio effect should have been found in the Experiment 2. Therefore, our results challenge the role of a mere SF processing since this information was cancelled out as cue for numerosity. In that respect, cancelling out the power spectrum information and still observing a SNA helps us to reject the BAFT account among the several proposed models in the literature, leaving open the possibility that the spatial–numerical link with nonsymbolic arrays of dots might emerge thanks to other cognitive factors (e.g., extraction of approximate numerical information and mapping across a spatially-oriented mental representation). These findings are in line with prior psychophysical studies showing that SF power spectrum *alone* cannot explain the typical behavioral effects observed in nonsymbolic numerical processing, such as the ratio dependence and scalar variability (e.g., Whalen et al., 1999), at least for moderate arrays of numerosity (Adriano et al., 2021a, b; Anobile et al., 2014; Anobile et al., 2017). Similarly, numerosity discrimination seems preserved also in animals when SFs are controlled in the numerical stimuli (Potrich et al., 2021). Indeed, the fact that power spectrum could be *correlated* with dot numerosity does not necessarily imply that the visual system takes into account *only* this feature to process numerosity magnitude (Wichmann et al., 2010). Accordingly, here, we clearly found that when we control for the (natural) correlation between global power spectrum and numerosity, the performance is not dramatically impaired (i.e., typical ratio effect is observed) and a clear SNA can therefore be found. In sum, these data indicate that SNAs emerging with nonsymbolic numerosities, at least in human adults, cannot be traced back to brain asymmetries relative to the processing of SFs power spectrum, leaving open the possibility that a combination of *attentional* and *numerical* factors could be at the origin of such effect and that cultural experiences may play a role in shaping this effect later in life (de Hevia, 2021).

Finally, our work is in line with studies reporting spatial–numerical associations in which numerosity (i.e., in the form of nonsymbolic arrays) was task relevant (Nemeh et al., 2018; Zhou et al., 2016), whereas there is little consensus when numerosity is not required to be explicitly estimated (e.g., numerical Posner-like task). Indeed, while some studies found that observing a relatively large numerical dot-array would accelerate saccades toward the subsequent target presented in the right space and vice versa for small numerical arrays (e.g., Bulf et al., 2016), others failed to find similar SNAs-like effects with both task-irrelevant symbolic and nonsymbolic stimuli (e.g., Cleland et al., 2020; Fattorini et al., 2015; see also Colling et al., 2020). The methodology we employed here can be easily integrated also with these paradigms to understand whether discrepancies, at least among studies using only dot arrays, might be due to SFs confounds, hence paving the way for further work aimed at understanding the origin of spatial–numerical associations.

## Supplementary Information

ESM 1 (DOCX 666 kb)

ESM 2 (CSV 2 kb)

ESM 3 (CSV 6 kb)

ESM 4 (CSV 7 kb)

ESM 5 (CSV 2 kb)

ESM 6 (CSV 6 kb)

ESM 7 (CSV 7 kb)

ESM 8 (DOCX 14 kb)

ESM 9 (RAR 159 kb)

ESM 10 (RAR 154 kb)

## Footnotes

1 Effect size specification option: as in Cohen (1988).

2 A total of 62 participants were originally tested. However, data were inspected before the analysis to verify that participants correctly understood the task. Consequently, 10 participants were discarded from the final sample because they presented an overall accuracy outside the ±1.5 interquartile range of the distribution.

3 The Greenhouse–Geisser epsilon (ε) correction for violation of sphericity was applied when needed, and original *F*, *df*, and corrected *p* values were reported.

4 A total of 60 participants were originally tested. Following data inspection, eight participants were discarded from the final sample because they presented an overall accuracy outside the ±1.5 interquartile range of the distribution.

5 We also run an overall ANOVA across the two experiments, hence including the type of stimulus (i.e., original vs. equalized) as a between-subjects factor. Results on RTs showed only a significant main effect of numerical ratio and, critically, of mapping. Furthermore, the congruency effect was not statistically different across the two experiments (original vs. equalized). See the Supplementary Materials for further information.
