# Supplementary material for: Spatial frequency equalization does not prevent spatial–numerical associations
Source: Psychon Bull Rev. 2022 Feb 7;29(4):1492–502. doi: 10.3758/s13423-022-02060-w (PMC8821778; doi:10.3758/s13423-022-02060-w)
Supplement: Supplementary file 1 — (DOCX 666 kb) [file 13423_2022_2060_MOESM1_ESM.docx]

**Supplementary Materials**

**Spatial frequency equalization does not prevent spatial-numerical associations**

Andrea Adriano^1*^, Luca Rinaldi^2, 3 #^, & Luisa Girelli^1, 4 #^

*^1^ Department of Psychology, University of Milano-Bicocca (Italy)*

*^2^ Department of Brain and Behavioral Sciences, University of Pavia, Pavia (Italy)*

*^3^ Cognitive Psychology Unit, IRCCS Mondino Foundation, Pavia (Italy)*

*^4^ NeuroMI, Milan Center for Neuroscience, Milano (Italy)*

*****Corresponding author: A. Adriano. Dipartimento di Psicologia, Università degli Studi di Milano-Bicocca, Piazza dell’Ateneo Nuovo 1, Edificio U6, 20126 Milano, Italy.

E-mail: [andrea.adriano@hotmail.com](file:///C:\Users\Andrea\Desktop\Papers\Paper%206\Submitted\andrea.adriano@hotmail.com)

^#^ These authors contributed equally to this work.

**Stimuli visual parameters**

Following the methodology of Gebuis and Reynvoet (2011), we analysed the visual cues for the stimuli in Experiment 1 using the data generated by their script. A regression analysis was run for each visual cue to check the relation between numerical distance and the different visual cues over the original stimuli. The dependent variable for the comparison task was the Weber fraction [(largest number – smallest number) / largest number] and the independent variable was the difference in visual properties of the two stimuli comprising each stimulus pair (Gebuis & Reynvoet, 2011). We reported the *R*^2^ and *p* values for the following visual cues: area extended (convex hull), total surface (the aggregate surface of all dots in one array), density (area extended/total surface), item size (average diameter of the dots presented in one array), and total circumference (circumference of all dots in one array, taken together).

Results showed that differences in area extended (*R*^2^ = .005, *p* = .45), density (*R*^2^ = .002, *p* = .65), total surface (*R*^2^ = .002, *p* = .62), item size (*R*^2^ = .0006, *p* = .80) and total circumference (*R*^2^ = .01, *p* = .28), did not reach the statistical significance and hence could explain only a very trivial (irrelevant) part of the variance in numerical distance.

**Figure S1:** Fourier amplitude spectrum displayed as polar plot for the Reference and the test stimulus with 8 items (ratio 0.66) or 10 items (ratio 0.8) as presented in the Experiment 1 (we reported this information only for these numerosities for reasons of space, but a similar pattern was found for all numerosities/ratios). Log-energy is plotted as a function of spatial frequency (distance from the origin; low-to-high) and orientation (angle).

**Supplementary Results: Experiment 1**

We ran two separate Bayesian repeated-measures ANOVAs (2 x 3) respectively on accuracy and RTs data, with mapping and ratio as independent variables. From an inspection of the table below (Table S1) for accuracy, the model that outperforms more the null model is the one with the main effect of ratio only, which received strong evidence in favor of the alternative hypothesis (*BF*_10_ > 100). We also compared the strength of the Bayes factor for the models that exclude or include the critical interaction term. The evidence *against* including the interaction is roughly a factor of 14, compared to the model with the main factors. This can be obtained as 3.75 × 10^44^ */* 2.75 × 10^43^ ≈ 14. Thus, the data are almost 14 times more likely under the two main effects model than under the full model (i.e., the one including also the interaction). Finally, the main effect of mapping received substantial support in favor of the null hypothesis (*BF*_10_ = 0.124). In sum, the Bayesian ANOVA for the accuracy reveals that the data provide very strong support for the main effect of ratio, as well as good evidence against mapping. The data also provide good evidence against including the interaction term.

The analysis of RTs revealed that the model that outperforms more the null model is the one with the main effect of ratio and mapping, and no interaction, which received strong evidence in favor of the alternative hypothesis (*BF*_10_ > 100) compared to all the other models (Table S2). Again, the evidence *against* including the interaction is almost a factor of 10, which can be obtained as 1.46 × 10^11^ */* 1.46 × 10^10^ ≈ 10. Thus, the data are almost 10 times more likely under the two main effects model than under the full model (i.e., the one including also the interaction).

| **Model Comparison** | | | | | | | | | | | |
| --- | --- | --- | --- | --- | --- | --- | --- | --- | --- | --- | --- |
| **Models** | | **P(M)** | | **P(M\|data)** | | **BF _M_** | | **BF _10_** | | **error %** | |
| Null model (incl. subject) |  | 0.200 |  | 2.949e -46 |  | 1.179e -45 |  | 1.000 |  |  |  |
| Ratio |  | 0.200 |  | 0.881 |  | 29.658 |  | 2.988e +45 |  | 1.221 |  |
| Mapping + Ratio |  | 0.200 |  | 0.111 |  | 0.498 |  | 3.755e +44 |  | 2.561 |  |
| Mapping + Ratio + Mapping  ✻  Ratio |  | 0.200 |  | 0.008 |  | 0.033 |  | 2.749e +43 |  | 4.844 |  |
| Mapping |  | 0.200 |  | 3.650e -47 |  | 1.460e -46 |  | 0.124 |  | 1.932 |  |
|  | | | | | | | | | | | |
| *Note.*  All models include subject | | | | | | | | | | | |

**Table S1.** Bayesian ANOVA on the Accuracy.

| **Model Comparison** | | | | | | | | | | | |
| --- | --- | --- | --- | --- | --- | --- | --- | --- | --- | --- | --- |
| **Models** | | **P(M)** | | **P(M\|data)** | | **BF _M_** | | **BF _10_** | | **error %** | |
| Null model (incl. subject) |  | 0.200 |  | 6.193e -12 |  | 2.477e -11 |  | 1.000 |  |  |  |
| Mapping + Ratio |  | 0.200 |  | 0.910 |  | 40.223 |  | 1.469e +11 |  | 1.605 |  |
| Mapping + Ratio + Mapping  ✻  Ratio |  | 0.200 |  | 0.090 |  | 0.398 |  | 1.460e +10 |  | 14.294 |  |
| Mapping |  | 0.200 |  | 3.181e  -6 |  | 1.272e  -5 |  | 513582.968 |  | 0.899 |  |
| Ratio |  | 0.200 |  | 2.612e  -7 |  | 1.045e  -6 |  | 42168.823 |  | 0.750 |  |
|  | | | | | | | | | | | |
| *Note.*  All models include subject | | | | | | | | | | | |

**Table S2.** Bayesian ANOVA on the RTs.


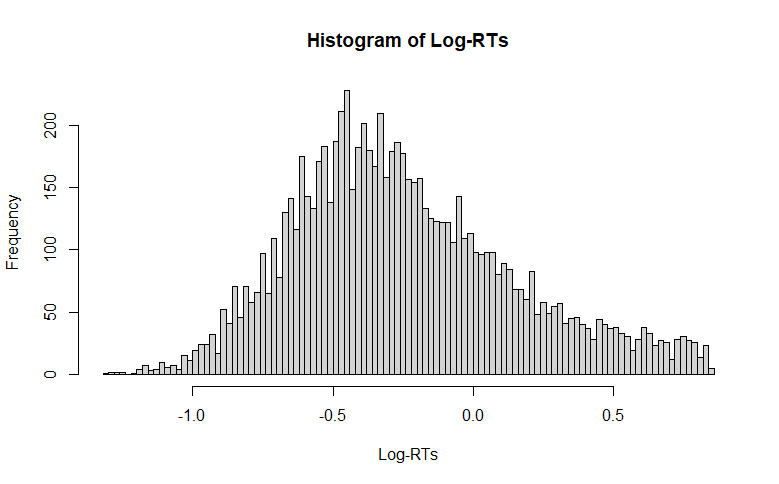


**Figure S2.** log-RTs distribution.

**Supplementary Results: Experiment 2**

As for the first experiment, we ran two separate Bayesian repeated-measures ANOVAs (2 x 3) respectively on accuracy and RTs data, with mapping and ratio as independent variables. As can be observed in the table below (Table S3), the model that outperforms more the null model is the one with the two main effects only, which received strong evidence in favor of the alternative hypothesis (*BF*_10_ > 100). We also compared the strength of the Bayes factor for the models that exclude or include the critical interaction term. The evidence *against* including the interaction is roughly a factor of 13.5, compared to the model with the main factors. This can be obtained as 3.75 × 10^44^ */* 2.75 × 10^43^ ≈ 14. Thus, the data are almost 13.5 times more likely under the two main effects model than under the full model (i.e., the one including also the interaction). In sum, the Bayesian ANOVA for the accuracy reveals that the data provide very strong support for the model with the two main effects. The data also provide good evidence against including the interaction term.

The analysis of RTs revealed that the model that outperforms more the null model is the one with the main effect of ratio and mapping, and no interaction, which received strong evidence in favor of the alternative hypothesis (*BF*_10_ > 100) compared to all the other models (Table S4). Again, the evidence *against* including the interaction is almost a factor of 12.5, which can be obtained as 1.027 × 10^7^ */* 821762.006 ≈ 12.5. Thus, the data are almost 12.5 times more likely under the two main effects model than under the full model (i.e., the one including also the interaction).

| **Model Comparison** | | | | | | | | | | | |
| --- | --- | --- | --- | --- | --- | --- | --- | --- | --- | --- | --- |
| **Models** | | **P(M)** | | **P(M\|data)** | | **BF _M_** | | **BF _10_** | | **error %** | |
| Null model (incl. subject) |  | 0.200 |  | 1.950e -58 |  | 7.801e -58 |  | 1.000 |  |  |  |
| Mapping + Ratio |  | 0.200 |  | 0.674 |  | 8.265 |  | 3.455e +57 |  | 1.649 |  |
| Ratio |  | 0.200 |  | 0.277 |  | 1.529 |  | 1.418e +57 |  | 0.869 |  |
| Mapping + Ratio + Mapping  ✻  Ratio |  | 0.200 |  | 0.050 |  | 0.208 |  | 2.540e +56 |  | 1.839 |  |
| Mapping |  | 0.200 |  | 6.812e -59 |  | 2.725e -58 |  | 0.349 |  | 1.680 |  |
|  | | | | | | | | | | | |
| *Note.*  All models include subject | | | | | | | | | | | |

**Table S3.** Bayesian ANOVA on the Accuracy.

| **Model Comparison** | | | | | | | | | | | |
| --- | --- | --- | --- | --- | --- | --- | --- | --- | --- | --- | --- |
| **Models** | | **P(M)** | | **P(M\|data)** | | **BF _M_** | | **BF _10_** | | **error %** | |
| Null model (incl. subject) |  | 0.200 |  | 8.950e -8 |  | 3.580e -7 |  | 1.000 |  |  |  |
| Mapping + Ratio |  | 0.200 |  | 0.919 |  | 45.660 |  | 1.027e +7 |  | 6.873 |  |
| Mapping + Ratio + Mapping  ✻  Ratio |  | 0.200 |  | 0.074 |  | 0.318 |  | 821762.006 |  | 2.337 |  |
| Ratio |  | 0.200 |  | 0.007 |  | 0.028 |  | 78158.146 |  | 1.545 |  |
| Mapping |  | 0.200 |  | 5.488e -6 |  | 2.195e -5 |  | 61.323 |  | 3.687 |  |
|  | | | | | | | | | | | |
| *Note.*  All models include subject | | | | | | | | | | | |

**Table S4.** Bayesian ANOVA on the RTs.


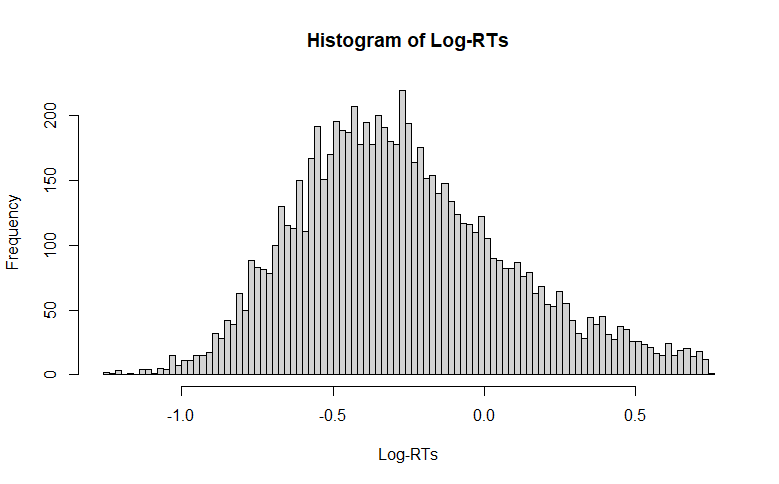
**Figure S3.** log-RTs distribution.

**Inter-experiments comparison**

As a further control of the analyses reported in the manuscript, we also run an inter-experiments comparison. In particular, we run two three-way mixed ANOVAs (2x2x3) with the Experiment (original vs equalized stimuli) as between-subjects variable, and the mapping (congruent vs incongruent) and the ratio (.66, .75, .8) as within-subjects variables, one over accuracy and one over correct RTs.

Results on accuracy data showed a significant main effect of experiment, *F*(1, 102) = 3.94, *p* = .05, η^2^_p_ = .037, suggesting a slightly higher accuracy with original stimuli as compared to the equalized ones. Furthermore, we found a significant effect of ratio, *F*(2, 204) = 393.01, ε = .89, *p* < .001, η^2^_p_ = .79, and a significant interaction between ratio and experiment, *F*(2, 204) = 5.55, ε = .91, *p* = .004, η^2^_p_ = .052, indicating that for higher ratios, discrimination became more challenging (i.e., more prone to errors) with equalized stimuli as compared to the original ones (see Figure S4A). The main effect of mapping and all the other interactions were not statistically significant (all *p* > .05). Bayesian analyses confirmed this pattern of results (Table S5), since the model that outperforms more the null model is the one containing the main effects of ratio and experiment and their interaction, which received strong evidence in favor of the alternative hypothesis (*BF*_10_ > 100).

Crucially, analysis of correct RTs (4% of data were discarded) revealed only a significant main effect of ratio, *F*(2, 204) = 76.63, ε = .88, *p* < .001, η^2^_p_ = .42, and a significant main effect of mapping, *F*(1, 102) = 15.087, *p* < .001, η^2^_p_ = .129 (see Figure S4B). The main effect of Experiment and all the other interactions were not statistically significant (all *p* > .05). Also in this case, Bayesian analysis confirmed this pattern of results (Table S6), since the model that outperforms more the null model is the one with the main effects of ratio and mapping (and no other interaction), which received strong evidence in favor of the alternative hypothesis (*BF*_10_ > 100). Furthermore, the main effect of experiment received anecdotal evidence for the null hypothesis (*BF*_10_ = .34), therefore suggesting that the effect of experiment was 3 times more likely under the null hypothesis (e.g., no difference).


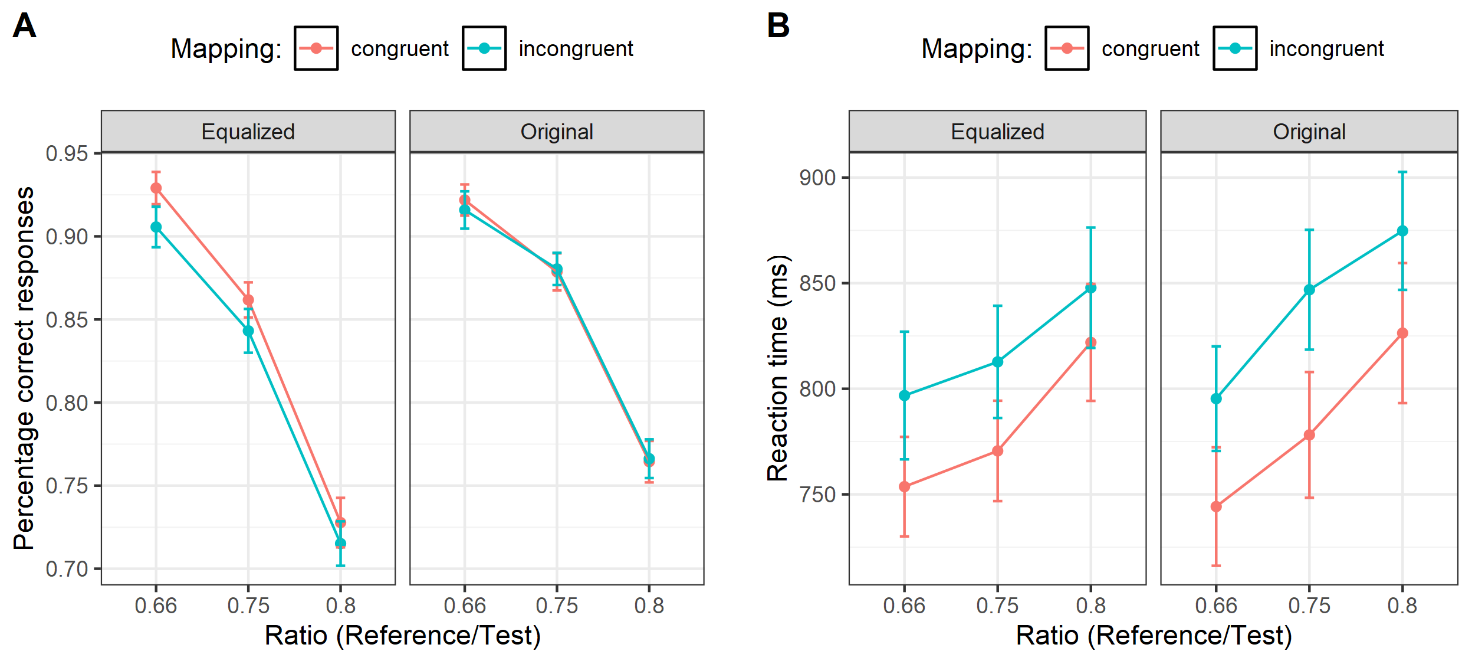


**Figure S4:** A) Percentage of correct responses as a function of the absolute ratio, the mapping condition and the Experiment (i.e., original vs. equalized stimuli). B) Reaction times as a function the absolute ratio, the mapping condition and the Experiment. Bars represent ±1 SEM.

| **Model Comparison** | | | | | | | | | | | |
| --- | --- | --- | --- | --- | --- | --- | --- | --- | --- | --- | --- |
| **Models** | | **P(M)** | | **P(M\|data)** | | **BF _M_** | | **BF _10_** | | **error %** | |
| Null model (incl. subject) |  | 0.053 |  | 6.197e -106 |  | 1.115e -104 |  | 1.000 |  |  |  |
| Ratio + Experiment + Ratio  ✻  Experiment |  | 0.053 |  | 0.446 |  | 14.497 |  | 7.199e +104 |  | 1.933 |  |
| Mapping + Ratio + Experiment + Ratio  ✻  Experiment |  | 0.053 |  | 0.225 |  | 5.235 |  | 3.636e +104 |  | 3.341 |  |
| Mapping + Ratio + Experiment + Mapping  ✻  Experiment + Ratio  ✻  Experiment |  | 0.053 |  | 0.125 |  | 2.561 |  | 2.010e +104 |  | 5.485 |  |
| Ratio + Experiment |  | 0.053 |  | 0.063 |  | 1.212 |  | 1.018e +104 |  | 5.280 |  |
| Ratio |  | 0.053 |  | 0.052 |  | 0.997 |  | 8.471e +103 |  | 1.565 |  |
| Mapping + Ratio + Experiment |  | 0.053 |  | 0.030 |  | 0.563 |  | 4.897e +103 |  | 3.281 |  |
| Mapping + Ratio |  | 0.053 |  | 0.024 |  | 0.447 |  | 3.912e +103 |  | 1.249 |  |
| Mapping + Ratio + Experiment + Mapping  ✻  Experiment |  | 0.053 |  | 0.015 |  | 0.272 |  | 2.400e +103 |  | 3.610 |  |
| Mapping + Ratio + Experiment + Mapping  ✻  Ratio + Ratio  ✻  Experiment |  | 0.053 |  | 0.010 |  | 0.189 |  | 1.680e +103 |  | 7.978 |  |
| Mapping + Ratio + Experiment + Mapping  ✻  Ratio + Mapping  ✻  Experiment + Ratio  ✻  Experiment |  | 0.053 |  | 0.005 |  | 0.091 |  | 8.137e +102 |  | 3.631 |  |
| Mapping + Ratio + Experiment + Mapping  ✻  Ratio |  | 0.053 |  | 0.001 |  | 0.025 |  | 2.267e +102 |  | 6.404 |  |
| Mapping + Ratio + Mapping  ✻  Ratio |  | 0.053 |  | 0.001 |  | 0.020 |  | 1.819e +102 |  | 2.187 |  |
| Mapping + Ratio + Experiment + Mapping  ✻  Ratio + Mapping  ✻  Experiment |  | 0.053 |  | 6.097e  -4 |  | 0.011 |  | 9.839e +101 |  | 2.111 |  |
| Mapping + Ratio + Experiment + Mapping  ✻  Ratio + Mapping  ✻  Experiment + Ratio  ✻  Experiment + Mapping  ✻  Ratio  ✻  Experiment |  | 0.053 |  | 4.080e  -4 |  | 0.007 |  | 6.584e +101 |  | 21.206 |  |
| Experiment |  | 0.053 |  | 4.841e -106 |  | 8.715e -105 |  | 0.781 |  | 2.290 |  |
| Mapping |  | 0.053 |  | 1.138e -106 |  | 2.048e -105 |  | 0.184 |  | 7.701 |  |
| Mapping + Experiment |  | 0.053 |  | 8.176e -107 |  | 1.472e -105 |  | 0.132 |  | 2.625 |  |
| Mapping + Experiment + Mapping  ✻  Experiment |  | 0.053 |  | 1.777e -107 |  | 3.199e -106 |  | 0.029 |  | 5.202 |  |
|  | | | | | | | | | | | |
| *Note.*  All models include subject | | | | | | | | | | | |

**Table S5.** Bayesian ANOVA on the Accuracy.

| **Model Comparison** | | | | | | | | | | | |
| --- | --- | --- | --- | --- | --- | --- | --- | --- | --- | --- | --- |
| **Models** | | **P(M)** | | **P(M\|data)** | | **BF _M_** | | **BF _10_** | | **error %** | |
| Null model (incl. subject) |  | 0.053 |  | 1.304e -20 |  | 2.346e -19 |  | 1.000 |  |  |  |
| Mapping + Ratio |  | 0.053 |  | 0.516 |  | 19.189 |  | 3.958e +19 |  | 4.047 |  |
| Mapping + Ratio + Experiment + Mapping  ✻  Experiment |  | 0.053 |  | 0.205 |  | 4.654 |  | 1.576e +19 |  | 5.490 |  |
| Mapping + Ratio + Experiment |  | 0.053 |  | 0.197 |  | 4.429 |  | 1.515e +19 |  | 4.825 |  |
| Mapping + Ratio + Mapping  ✻  Ratio |  | 0.053 |  | 0.027 |  | 0.496 |  | 2.056e +18 |  | 1.842 |  |
| Mapping + Ratio + Experiment + Ratio  ✻  Experiment |  | 0.053 |  | 0.016 |  | 0.300 |  | 1.260e +18 |  | 7.308 |  |
| Mapping + Ratio + Experiment + Mapping  ✻  Experiment + Ratio  ✻  Experiment |  | 0.053 |  | 0.016 |  | 0.292 |  | 1.226e +18 |  | 10.514 |  |
| Mapping + Ratio + Experiment + Mapping  ✻  Ratio + Mapping  ✻  Experiment |  | 0.053 |  | 0.012 |  | 0.210 |  | 8.865e +17 |  | 9.602 |  |
| Mapping + Ratio + Experiment + Mapping  ✻  Ratio |  | 0.053 |  | 0.008 |  | 0.152 |  | 6.416e +17 |  | 7.694 |  |
| Mapping + Ratio + Experiment + Mapping  ✻  Ratio + Mapping  ✻  Experiment + Ratio  ✻  Experiment |  | 0.053 |  | 0.001 |  | 0.019 |  | 7.960e +16 |  | 15.522 |  |
| Mapping + Ratio + Experiment + Mapping  ✻  Ratio + Ratio  ✻  Experiment |  | 0.053 |  | 8.925e  -4 |  | 0.016 |  | 6.846e +16 |  | 10.086 |  |
| Mapping + Ratio + Experiment + Mapping  ✻  Ratio + Mapping  ✻  Experiment + Ratio  ✻  Experiment + Mapping  ✻  Ratio  ✻  Experiment |  | 0.053 |  | 3.988e  -5 |  | 7.179e  -4 |  | 3.059e +15 |  | 14.756 |  |
| Ratio |  | 0.053 |  | 3.127e -10 |  | 5.628e  -9 |  | 2.399e +10 |  | 0.636 |  |
| Ratio + Experiment |  | 0.053 |  | 1.128e -10 |  | 2.030e  -9 |  | 8.650e  +9 |  | 4.328 |  |
| Ratio + Experiment + Ratio  ✻  Experiment |  | 0.053 |  | 9.070e -12 |  | 1.633e -10 |  | 6.958e  +8 |  | 6.787 |  |
| Mapping |  | 0.053 |  | 1.578e -12 |  | 2.840e -11 |  | 1.210e  +8 |  | 0.980 |  |
| Mapping + Experiment |  | 0.053 |  | 5.853e -13 |  | 1.054e -11 |  | 4.490e  +7 |  | 3.849 |  |
| Mapping + Experiment + Mapping  ✻  Experiment |  | 0.053 |  | 4.924e -13 |  | 8.864e -12 |  | 3.777e  +7 |  | 4.525 |  |
| Experiment |  | 0.053 |  | 4.460e -21 |  | 8.028e -20 |  | 0.342 |  | 3.447 |  |
|  | | | | | | | | | | | |
| *Note.*  All models include subject | | | | | | | | | | | |

**Table S6.** Bayesian ANOVA on the RTs.

**Individual Regression Coefficients**

To further corroborate the regression analyses, we also ran for each experiment an analysis of the individual slopes. Individual data were fitted with a regression model as we did at the group level, this time on each participant (i.e., including the difference between RTs with the right and left hands as dependent variable, and numerosity as predictor). Individual regression coefficients for each subject were thus computed and entered in a one-sample *t*-test to verify whether the individual slopes deviate from zero. Results showed that in both Experiment 1, *t*(51) = -3.27, *p* = .001, *d* = .45, and Experiment 2, *t*(51) = -2.29, *p* = .025, *d* = .31, overall, the regression coefficients were negative and significantly different from zero.
