# Supplementary material for: Spatial frequency equalization does not prevent spatial–numerical associations
Source: Psychon Bull Rev. 2022 Feb 7;29(4):1492–502. doi: 10.3758/s13423-022-02060-w (PMC8821778; doi:10.3758/s13423-022-02060-w)
Supplement: Supplementary file 8 — (DOCX 14 kb) [file 13423_2022_2060_MOESM8_ESM.docx]

**METADATA**

**“Exp1_Accuracy.csv” and “Exp2_Accuracy.csv”**

*Participant:* indicates the number of assigned to each participant.

*Congruent.0.66:* indicates the accuracy for the Mapping condition and the Ratio between numerosities.

*Congruent.0.75:* indicates the accuracy for the Mapping condition and the Ratio between numerosities.

*Congruent.0.8:* indicates the accuracy for the Mapping condition and the Ratio between numerosities.

*Incongruent.0.66:* indicates the accuracy for the Mapping condition and the Ratio between numerosities.

*Incongruent.0.75:* indicates the accuracy for the Mapping condition and the Ratio between numerosities.

*Incongruent.0.8:* indicates the accuracy for the Mapping condition and the Ratio between numerosities.

**“Exp1_RTs.csv” and “Exp2_RTs.csv”**

*Participant:* indicates the number of assigned to each participant.

*Congruent.0.66:* indicates the log-RTs for the Mapping condition and the Ratio between numerosities.

*Congruent.0.75:* indicates the log-RTs for the Mapping condition and the Ratio between numerosities.

*Congruent.0.8:* indicates the log-RTs for the Mapping condition and the Ratio between numerosities.

*Incongruent.0.66:* indicates the log-RTs for the Mapping condition and the Ratio between numerosities.

*Incongruent.0.75:* indicates the log-RTs for the Mapping condition and the Ratio between numerosities.

*Incongruent.0.8:* indicates the log-RTs for the Mapping condition and the Ratio between numerosities.

**“Exp1_RTs-diff.csv” and “Exp2_RTs-diff.csv”**

*Participant:* indicates the number of assigned to each participant.

*Condition:* indicates the Numerosity in the test stimulus.

*Par:* indicates the difference in log-RTs between right-hand and left-hand for a given numerosity in test stimulus.
